# Skewed sex ratios and violence against women in Pakistan

**DOI:** 10.1017/ehs.2025.10003

**Published:** 2025-07-01

**Authors:** Olympia L. K. Campbell, Maheen Pracha, Ruth Mace

**Affiliations:** 1 Institute for Advanced Study in Toulouse, University of Toulouse Capitole, Toulouse, France; 2 Human Rights Commission of Pakistan, Lahore, Pakistan; 3 Department of Anthropology, University College London, London, UK

**Keywords:** sex ratio, violence against women, suicide, Pakistan, newspaper reports

## Abstract

Concerns have been raised that an excess of men leads to societal violence, including violence against women, although recent evidence has challenged this view. One area that remains untested is honour killings, a type of femicide perpetrated by unrelated family members, such as intimate partners, and related family members, such as parents and siblings. Using a novel data set of media reports of honour killings from Pakistan we test whether the sex ratio is associated with femicide. To address reporting bias, we implement two case-control studies. The first compares media reports of honour killings to male suicides. The second compares honour killings perpetrated by unrelated individuals to those perpetrated by kin. We find evidence that honour killings perpetrated by unrelated individuals are higher in male-biased areas compared to those perpetrated by kin. Honour killings of women by kin therefore appear less sensitive to the sex ratio. Results align with sexual selection theory, suggesting more male competition may lead to more violence. We also find weak evidence that male-biased areas report more male suicides than honour killings. However, we caution against drawing causal conclusions due to potential confounding variables, particularly economic deprivation. This highlights the challenges of studying sensitive topics quantitatively.

## Graphical abstract

**Figure:**
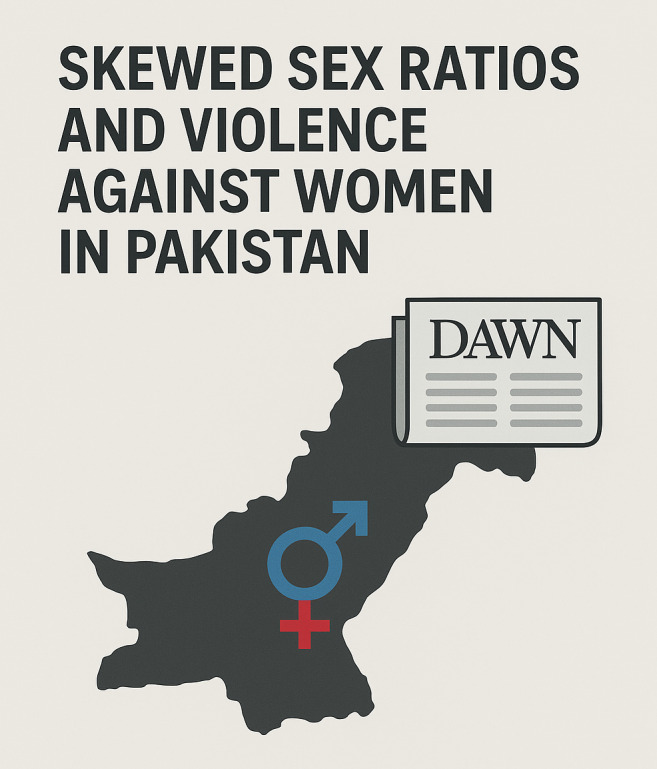

## Introduction

Over the years, imbalanced sex ratios, especially an excess of men, have raised concerns about potential negative societal effects, such as increased violence against women (Hudson & den Boer, 2004). However, others have suggested instead that male-biased sex ratios may actually reduce violence (Kokko & Jennions, 2008). Although prior studies have explored associations between the sex ratio and sexual and intimate partner violence, none have explored their effect on honour-based violence, a type of violence often carried out by blood relatives in the name of honour.

Sexual selection and parental investment theory (Trivers, 1972) predicted that the sex with greater reproductive potential – typically males – would compete more for mates (Bateman, 1948; Janicke et al., 2016). A male-skewed sex ratio is expected to intensify this competition, as an excess of males compete for a limited number of females. This competition is often considered to be aggressive, showing up as increased violent crime within human society. In line with these predictions the strength of sexual selection increases weaponry in animals (Bro-Jørgensen, 2007) and in humans, male-biased sex ratios have been found to associate with total murder rates (Drèze & Khera, 2000), crime (Edlund et al., 2013), male-on-male violence (Filser et al., 2021), femicide (Amaral & Bhalotra, 2017; Titterington, 2006), intimate-partner violence (D’Alessio & Stolzenberg, 2010) and aggression and depression in men (Zhou & Hesketh, 2017).

Conversely, newer models in sexual selection and marriage market theory in human societies suggest male abundance can lead to less violent competition and higher provisioning of care in males, as males seek to improve their desirability or avoid being returned to a mating pool where females are in short supply (Angrist, 2002; Kokko & Jennions, 2008). Supporting this, male-biased adult sex ratios correlate with increased paternal care in birds (Liker et al., 2013) and higher rates of fathers cohabiting with mothers in humans (Schacht & Kramer, 2016; Uggla & Mace, 2017). The rarer sex is also predicted to have higher bargaining power and the ability to demand greater levels of a desirable trait, with the opposite sex responding to this choice. Higher overall rates of marriage in male-biased areas of the USA and Australia (Angrist, 2002; Grosjean & Khattar, 2019) as well as lower rates of marriage among poorer men (Pollet & Nettle, 2008) have been interpreted as female choice prompting men to make stronger financial and emotional commitments, whereas those that cannot remain unmarried. By contrast, in female-biased areas, men may invest less in relationships and more in mating effort, leading to more promiscuity, lower cohabitation (Uggla & Mace, 2017), higher divorce rates (Uggla & Andersson, 2018), and earlier ages at first birth, reflective of lower female bargaining power (Uggla & Mace, 2016). Analogously, in birds, higher rates of short-term pair bonds are observed where females outnumber males (Liker et al., 2014).

Regarding violence against women specifically, male-biased areas are often predicted to show higher levels of intimate-partner and sexual violence. Scarcity of women may lead men to engage in mate-guarding through intimate-partner violence that prevents women from leaving a relationship. Positive associations between male-biased sex ratios and intimate-partner violence have been found in the USA and six Asian countries (D’Alessio & Stolzenberg, 2010; Diamond-Smith et al., 2018). However, others have proposed that intimate-partner violence will be lower in male-biased areas, as men invest in wealth accumulation and caring qualities that increase their attractiveness as partners (Schacht et al., 2014). In line with this, marital satisfaction and happiness has been found to be higher in historically male-biased areas of Australia (Grosjean & Brooks, 2017). Additionally, it has been suggested that men unable to secure partners might resort to coercion and rape. Studies linking the sex ratio and sexual violence in humans is mixed with evidence of positive (Amaral & Bhalotra, 2017; Baranov et al., 2023; Barber, 2000), negative (Obrien, 1991), and U-shaped (Pabst et al., 2022) relationships.

That the adult sex ratio has been found to associate with both violence against women and male commitment, paternal care, and marital satisfaction is not contradictory. Expecting a consistent association between the sex ratio and negative outcomes for women involves several assumptions that are unlikely to hold. First, it assumes that male–male competition for women is often violent, and second, that all men behave in the same way. This is clearly not the case. In many cases, competition will be expressed in non-violent terms, such as through competition for wealth. Additionally, the behaviours of partnered and unpartnered men, as well as those of richer and poorer men, are likely to differ. While one man may engage in wealth accumulation and paternal investment, another may engage in mate guarding and violence, and the likelihood that either occurs will depend on the particular ecological and cultural context.

However, mixed results may also be a product of using sex-ratio data at high levels of aggregation, which can produce the opposite association to what would be found at a lower level of aggregation level (Filser et al., 2021; Pollet et al., 2017). Crime data are also often used in aggregate, preventing researchers from examining how individual-level factors affect an association between sex ratios and violence. Filser et al. (2021) are notable for using longitudinal individual-level data from Sweden, from which they find a positive association between sex ratios and violence. However, they highlight that concerns remain given that the sex ratio also associates with non-violent crime and violent crime from women, which is not predicted, indicating that there are still uncontrolled confounders.

One area of violence that remains undertheorized and underexplored in the sex ratio literature is honour-based violence (HBV), defined as violence perpetrated in response to threats to one’s honour. In honour cultures common to the Middle East and South Asia, such as Pakistan (the focus of this study), a family’s honour is strongly affected by the behaviour of the women, particularly in relation to her sexual and marital choices (Gill et al., 2014; Sev’er & Yurdakul, 2016). HBV in this context includes restricting female mobility, male chaperones, female claustration, physical violence, and, at its most extreme, honour killings. Honour killings, a type of femicide, typically follow accusations of sexual impropriety, including the rejection of arranged marriages, premarital sex, adultery, and lesser infractions such as unchaperoned outings or wearing makeup and clothes deemed immodest (Gill et al., 2014). Overall, The United Nations Population Fund estimates that there are 5000 honour killings occurring annually (UNFPA, 2000). Victims tend to be young and of reproductive age and honour killings are often, but not always, perpetrated by male blood relatives, such as fathers, brothers, uncles, and cousins (D’Lima et al., 2020; Khan, 2006; Kressel et al., 1981; Kulczycki & Windle, 2011; Kulwicki, 2002). In this sense honour killings are fundamentally distinct from other forms of violence against women that are perpetrated by unrelated men, such as intimate partners or unrelated perpetrators of sexual violence. From a kin selection perspective this is an evolutionary puzzle. Harming your kin is costly to your inclusive fitness, and particularly costly in the case of harming and killing a female relative of reproductive age.

We anticipate that honour killings should respond to the sex ratio. Specifically, we hypothesize that a male-biased sex ratio will be negatively associated with honour killings. This could occur for two reasons. First, female bargaining power and status could increase, relaxing proscriptions around female behaviour and reducing the number of honour killings. Or the increased ‘value’ of women may dissuade family members from committing honour killings, particularly when in-demand daughters can enable them to secure advantageous marital alliances, demand reduced dowries, or command higher bride prices (Miller, 2001). Not just parents but brothers too will have a vested interest in the marriage of their sisters, particularly in contexts where exchange marriage is practised, such as Pakistan’s watta satta practise, where sets of brothers exchange sisters as wives (Jacoby & Mansuri, 2010). Indeed, in some parts of Pakistan, particularly rural Sindh, the majority of marriages are exchange marriages (Rehan & Qayyum, 2017). In areas of female scarcity, there may be competition among brothers for watta satta marriages, as has been documented in other forms of exchange marriage (Chagnon et al., 2017), generating a strong incentive to ensure the well-being of their sisters. These two lines of reasoning differ in whether female scarcity ultimately benefits the status of women.

Many femicides that are perpetrated by intimate partners will also be described as honour killings in Pakistan (Nasrullah et al., 2009), even though such femicide is common worldwide where they are generally not described as honour killings (Campbell et al., 2007). These would not be predicted to follow the same sex ratio patterning as kin-perpetrated honour killings. Rather, they may follow similar patterning to intimate-partner violence, given that femicide often follows previous domestic abuse or estrangement from a partner (Campbell et al., 2007). Similar to intimate-partner violence there is evidence for higher rates of femicide in male-biased areas, although this research is unable to differentiate among kin and non-kin perpetrators (Amaral & Bhalotra, 2017; Titterington, 2006).

It is also important to note that using an evolutionary cost–benefit framework does not imply that we believe that women are or should be treated as commodities. Nor, by any means, are evolutionary explanations the only or key drivers of violence in society. Cultural context and power dynamics within a society will greatly affect the ability of men to engage in violence and of women to report it. Nonetheless, evolutionary and economic costs and benefits, and how such pressures influence societal phenomena, can have broad explanatory power that may be necessary to understanding the underlying ecological factors that drive violence against women.

Honour killings remain quantitatively understudied, likely because of a lack of data sets large enough for analysis across geographic areas. Given the sensitivity surrounding honour killings due to their violence, their illegality and their occurrence within close-knit familial circles, obtaining accurate estimates of their prevalence remains difficult. Conducting interviews on honour killings is subject to many constraints. Individuals are unlikely to admit to knowing that a serious crime has occurred and there would be ethical restrictions to conducting interviews, particularly related to disclosing information to police if individuals admit to honour killings. Nor do police and governments routinely collect reliable data on honour killings. One means by which estimates of honour killings can be calculated is via media reports.

Media data have been central to research on collective action and protest movements and are common in political science and economics (Earl et al., 2004). However, they suffer from selection bias as newspapers do not report on all occurrences of an event nor can they, as many events will go unreported, particularly in the case of violence against women. Selection is affected by the ‘newsworthiness’ of the event, which can be influenced by the proximity of the event to the news agency and the extremeness of the event, such as how violent it was (Johnstone et al., 1994). Additionally, in the case of honour killings and other crimes, it is affected by the routines and ‘beats’ of crime reporters and the extent to which the public is interested in a particular crime. Attention cycles and the political climate can also strongly influence the likelihood of coverage. For example, high-profile cases, such as the 2016 honour killing of Qandeel Baloch, a Pakistani social media influencer, likely influences public interest and the likelihood of further cases being covered. The issue of selection bias in media reports is sizeable (see Ortiz et al., 2006 for a detailed discussion); however, they are one of the only means by which to study the topic.

Here, we use a data set of media reports of honour killings collected by the Independent Human Rights Commission of Pakistan from 2015 to 2022 to test whether the district-level sex ratio is associated with reports of honour killings. In order to address selection bias, we employ two simplistic case-control studies. The first compares all reports of honour killings/femicide to an identically collected data set on male suicide, which acts as the control group. The second divides the honour killings database into honour killings perpetrated by kin and those perpetrated by unrelated individuals, such as intimate partners, in-laws, and neighbours.

## Methods

### Data

Data on honour killings and male suicide is sourced from the Human Rights Commission of Pakistan (HRCP), who systematically compile data on human rights abuses reported in the press, including honour killings and suicide. These data have been digitized since 2015 and we use data from 2015 to 2022. The sampling procedure is as follows. Employees of the HRCP located in nine offices across Pakistan scan a list of approximately 26 newspapers (Table S1) each week and search for keywords relating to human rights abuses (see Table S2 for keywords relating to our variables of interest). To be defined as an honour killing the newspaper article must mention the concept of honour or the term karo-kari, which is commonly used as a synonym for honour killing, particularly in the province of Sindh.

The choice of newspapers is decided by employees of the HRCP. All major English-language newspapers are covered whereas many local Urdu ones are not, as they are not deemed credible enough (personal correspondence with the HRCP). Newspapers regularly report the occurrence of honour killings and suicides, but reports vary in their detail. Each case is coded for key variables including city, province, number of victims, sex of victim, and relationship between victim and perpetrator in the case of honour killings. The data sets are checked by the HRCP for duplicated reports.

In order to assign each report to a district, we used the *tmap* package in R to geocode the city using the OpenStreetMap Nominatim (Tennekes, 2018). The geocoded latitude and longitudes were then mapped to a district using shapefiles of administrative regions of Pakistan downloaded from The Humanitarian Data Exchange (data.humdata.org) and the *sf* package in R (Pebesma, 2018). Administratively, Pakistan is divided into six provinces, 160 divisions and 577 districts. The six provinces include Punjab, Sindh, Khyber Pakhtunkhwa, Baluchistan, Islamabad Capital, Gilgit Baltistan, and Azad Kashmir. Islamabad, the capital of Pakistan, is its own province, but we subsume it into the division of Rawalpindi and province of Punjab, as Islamabad only has one district and therefore contains no variation in our district-level variables.

Data on district-level variables were taken from the Pakistan Census, conducted in 2017. We calculate the district-level adult sex ratio of those aged 15–49 by dividing the number of men by the number of women and multiplying by 100 to give a ratio of the number of men per 100 women. We use ages 15–49 as this contains the individuals most likely involved in the marriage market as well as the age group of women that tend to be most at risk of violence (Murshid & Critelli, 2020; Wilson et al., 1995). Other variables include the district-level proportion of property that is owned by women, the sex difference in literacy and the number of police stations at the division level. All data can be found on the Pakistan Bureau of Statistics website (pbs.gov.pk). Night-time luminosity was taken from the geographic covariates available from the 2018 Demographic Health Survey (DHS) for Pakistan (available at DHSprogram.com) and a district-level average was calculated by assigning the DHS clusters to districts and taking the average.

### Analytic design

#### Reporting bias

Newspaper report data suffer from a high degree of reporting bias, creating selection bias, which occurs when the individuals within a data set differ systematically from the population of interest. In our case, a large proportion of the variation in our data set will represent variation in reporting an honour killing or suicide, rather than variation in the rate of occurrence. For example, in the whole data set of reported honour killings from 2015 to 2022 only seven cases were reported in the province of Gilgit Baltistan compared to 5166 in Punjab (Table S3), most likely reflecting Gilgit Baltistan’s remoteness and lower economic development. Similarly, the district-level number of reports of honour killings and male suicide are highly correlated (Figure S1). Because of this we cannot accurately estimate either the geographic distribution of honour killings or the association with district-level sex ratio.

To address this, we implement two case-control studies, a method commonly used in the medical sciences, where controls are chosen to be representative of the population which produced the cases. The first uses all reported honour killings where at least one woman was the victim regardless of perpetrator as the cases and male suicides as the controls. Grouping honour killings like this regardless of perpetrator allows us to include those reports where perpetrator information was missing. One can consider it as a measure of overall reports of femicide. We exclude all reports of honour killings where only men were victims and all reports of female suicide. As the screening method implemented by the HRCP between honour killings and suicides is the same, the hypothetical ‘population’ that produced both data sets is also the same. The difference between the cases and controls should therefore represent a meaningful difference in the number of reports of each type in a particular district. Key drivers of selection bias that should affect both equally are media presence, police presence, population size and general development of the area (Ortiz et al., 2006).

We acknowledge that there will be selection bias that differs between reporting suicides and honour killings. For example, reporting of violence against women may be affected by the gender equality norms of the area, with more gender-equal areas being more likely to report cases due to awareness raising and higher prosecution rates. On the other hand, this may also affect the actual baseline rate of honour killings, if more gender-equal areas are less likely to commit them. To control for this, we use proxy measures of gender equality including the sex difference in literacy and the proportion of property that is owned by women. Furthermore, for an association to be meaningful, suicide should not be related to our exposure of interest, the sex ratio. However, there is evidence that the sex ratio is associated with the male suicide rate, often considered to be mediated by the marriage market, with an overabundance of men leading to increased male singledom and depression (Baranov et al., 2023; Kuroki, 2014; Snopkowski & Turner, 2023). One alternative would be to use only cases of female suicide, but this is problematic as in Pakistan female suicides can be related to domestic violence and the label is used to conceal honour killings (Patel & Gadit, 2008).

The second case-control study compares honour killings to themselves, by defining those perpetrated by kin as cases and those perpetrated by non-kin as controls. In this comparison the selection bias affecting the two groups should be extremely similar. In both these model specifications it is possible that the control group drives any association with sex ratio that we find. To understand whether it is the cases or controls, we plot the raw numbers of reports of both against the sex ratio.

We also include controls that we believe are associated with selection bias, particularly those related to how well developed an area is and therefore how well serviced it is by police and media. These include night-time luminosity, urbanization, population size, and number of police stations. Broadly speaking, controlling for an ancestor of the outcome, if they do not open any confounding paths, can help to reduce variation in the outcome variable thus improving the precision of estimates (Cinelli et al., 2022).

#### Confounders

What drives skewed sex ratios could confound any relationship we find. Skewed sex ratios can be driven by three things, (1) sex selective abortion, (2) differential mortality rates and (3) differential migration patterns. In Pakistan, although there is a strong preference for sons, there is little evidence of sex-selective abortion; instead parents opt to continue having children until the desired number of sons is reached (Javed & Mughal, 2022; Zaidi & Morgan, 2016). Mortality rates do not appear to differ between men and women in Pakistan as a whole, although this may well belie regional variation (Pakistan Bureau of Statistics, 2022). Migration in the country is high, with 14 per cent of households having at least one member who has migrated to another district, with women most often migrating for marriage whereas men most often migrate for economic opportunity (NIPS/Pakistan, N.I. of P.S.-, ICF, 2019). Pearson’s correlation between the district-level sex ratio and the percentage of the population living in an urban area is 0.53 (95% CI = 0.34–0.67) indicating that male migration into urban areas could be driving a significant portion of the sex ratio skew (Figure S2). Overall, any variation in sex ratio is likely to be driven by the last two reasons. One possible confounding path is through gender equality, which can affect both differential mortality rates and the occurrence of violence against women. A second confounding path likely exists through the economic development of an area, which can affect both sex specific migration and violence against women, with some evidence that migration is a risk factor for intimate-partner violence (Terrazas & Blitchtein, 2022). We attempt to control for these confounding paths through our proxy measures of gender equality and through the night-time luminosity and urbanization of an area as ancestral causes of migration.

#### Statistical analyses

We fitted logistic mixed-effect models with binomial error distributions in R using the function *glmer* implemented in *lme4* (Bates et al., 2015). We conduct two main analyses; the first includes all honour killings with at least one female victim (cases) and male suicides (controls) and the second includes honour killings perpetrated by kin (cases) and those perpetrated by unrelated individuals (controls). A case is given a value of 1 and a control a value of 0. We included random intercepts for district, division and province to account for the geographic clustering of our data set. Due to the very different measurement scales of our variables, all variables are scaled by dividing them by their standard deviation using the function *scale*. Thus, each odds ratio should be interpreted as the impact of a one standard deviation increase.

We include only districts that recorded at least 20 combined reports of male suicides and honour killings for the first analysis, and 20 reports of honour killings in which the relationship between the victim and perpetrator was known for the second analysis. This is because districts that reported fewer than 20 reports tend to exhibit more extreme ratios of cases to controls, potentially biasing results. This results in only analysing districts within the provinces of Punjab, Sindh, Balochistan and Khyber Pakhtunkhwa, as too few cases were reported from Gilgit Baltistan, Azad Kashmir and the Federally Administered Tribal Areas to examine variation across their districts (Table S3).

The two main analyses combine data from the years 2015–2022 but we only have sex ratio data for the year 2017. Thus, we implicitly assume that the sex ratio did not vary considerably between these years. To address this, we conduct an additional analysis that restricts our data set to only reports from 2017 and we apply the same 20 report minimum described above. Some districts exhibit potentially implausibly large differences in the relative numbers of cases and controls. We conduct a robustness check that removes these outlier districts. We define outliers as districts where the district-level proportion of male suicides (male suicides/male suicides + all honour killings for Model 1) and non-kin perpetrated honour killings (non-kin/non-kin + kin perpetrated honour killings for Model 2) is either greater than 0.85 or less than 0.15.

**Figure 1.**
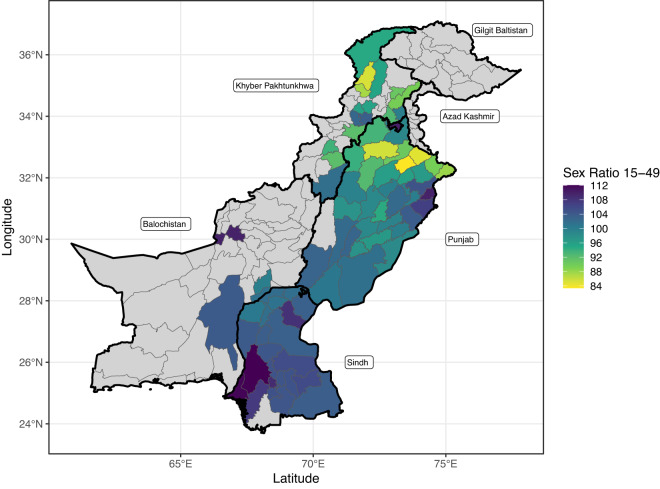
The sex ratio (number of men per 100 women) of individuals aged 15–49 among districts where at least 20 combined reports of honour killings and male suicides were reported. Sex ratios above 100 indicate an excess of men, whereas those below 100 indicate an excess of women. Grey districts are those which did not meet this minimum threshold of reports. Thick black lines denote the outline of the six provinces. Thin grey lines denote the outlines of districts.

Our main sample consists of 79 districts that reported at least 20 combined reports of honour killings with at least one female victim and male suicides. These districts exhibit an extremely wide range of adult sex ratios aged 15–49 from 83.42 to 112.03 (Figure 1) with a mean of 99.39 and a standard deviation of 6.54. The overall population size across the districts for adults aged 15–49 ranges from 44,191 to 8,614,237.

## Results

Between 2015 and 2022 a total of 8312 cases of honour killings were reported in the media across Sindh, Punjab, Balochistan and Khyber Pakhtunkhwa. Of these there were 7097 with only female victims, 542 with only male victims, 634 with victims of both sexes, and 39 where the sex of the victim is not recorded. Of those with at least one female victim, the majority perpetrator was a husband/ex, responsible for 31 per cent of honour killings (Table 1). Relatives were responsible for 30% of the cases, of whom siblings were the most common perpetrator. Of those with only male victims the majority perpetrator was part of the ‘others’ group including acquaintances, neighbours and employers, responsible for 52 per cent. Relatives were responsible for 17 per cent. For the remainder of the paper when we refer to honour killings we are only referring to those with at least one female victim.

**Table 1.** Number of media reports of honour killings by perpetrator in the four provinces of Punjab, sindh, balochistan, and khyber pakhtunkhwa

	Perpetrator of honour killing								
	Husband/ex	In-laws	Parents	Siblings	Son/daughter	Other relative	Others including acquaintance/neighbour/employer	Not known	Total
At least one female victim	2373 (31%)	538 (7%)	572 (7%)	1000 (13%)	232 (3%)	533 (7%)	1025 (13%)	1458 (19%)	7731 (100%)
Only male victims	7 (1%)	45 (8%)	7 (1%)	17 (3%)	2 (<1%)	73 (13%)	284 (52%)	107 (20%)	542 (100%)

**Figure 2.**
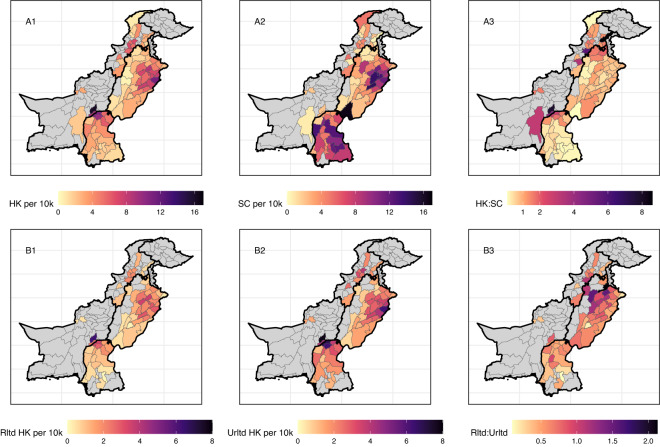
Heat maps of reports. Row A presents the number of honour killings (A1) and male suicides (A2) per 10,000 people that were reported and the ratio between the two (A3). Row B presents the number of honour killings perpetrated by kin (B1) and non-kin (B2) per 10,000 people and the ratio between the two (B3). Only districts where a minimum number of 20 combined reports of honour killings and suicide are included for row A. The same minimum threshold is applied for row B but with a 20-report minimum for honour killing reports where the perpetrator is known. Grey districts are those that did not meet these thresholds. Thick black lines denote the province boundaries. Note the different scales for rows A and B.

Figure 1 presents a heat map of the adult sex ratios for the districts in our sample and Figure 2 presents the number of femicide and male suicide reports. In terms of raw reporting adjusting for population size, the north-east of Punjab emerges as a hotspot for both honour killings and male suicides (A1 and A2). Southern Sindh has a high number of male suicides relative to honour killings, while to the north of Punjab and into Khyber Pakhtunkhwa there are more honour killings (A3). Examining the ratio of honour killings perpetrated by related kin to those perpetrated by unrelated individuals shows a slight excess of honour killings perpetrated by kin across the north of Punjab (B3).

**Figure 3.**
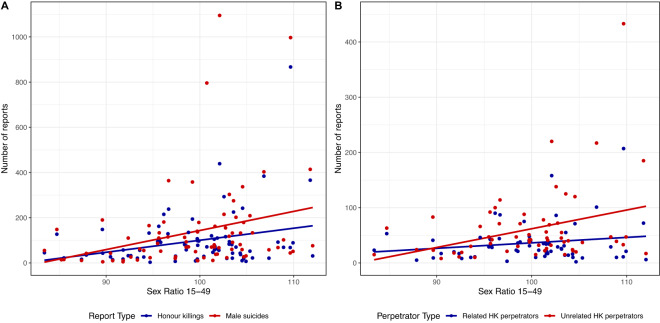
Regression plots showing the relationship between raw number of reports per district of (A) all honour killings and male suicides and (B) honour killings perpetrated by relatives and those perpetrated by non-relatives, and the district-level sex ratio 15–49 (number of men per 100 women). The difference between the slopes of the lines gives us an idea of which group is driving the associations in our analyses.

Mixed-effect logistic regressions indicate that there is an association between the sex ratio and either honour killings or male suicide (Model 1: OR = 0.74, 95% CI = 0.55–1.00, *p* < 0.1). To understand whether this is being driven by a negative relationship with honour killings, a positive relationship with male suicides, or both, we plot the raw reporting data against the sex ratio (Figure 3, panel A). The steeper slope for male suicides indicates that what is driving the relationship in Model 1 is a stronger positive relationship between an increasingly male-biased sex ratio and male suicides compared to honour killings. This does not preclude that honour killings may independently be responsive to the sex ratio and indeed the slope also appears positive. However, it does imply that, counter to our predictions, it is in fact male suicide that is responsive to the sex ratio, to a greater degree than honour killings, with more suicides occurring in more male-biased areas.

Separating honour killings into those perpetrated by kin and those perpetrated by non-kin and plotting the raw data, we find that more non-kin perpetrated honour killings/femicides are reported in areas of male excess, compared to those perpetrated by kin (Figure 3 panel B and Table 2 Model 2). By transforming the odds ratio, we can say that an honour killing perpetrated by non-kin is 1.26 (95% CI 1.08–1.47, *p* < 0.01) times more likely to be reported when the sex ratio increases by 1 standard deviation, compared to a kin-perpetrated honour killing.

**Table 2.** Multi-level logistic regression models. Model 1 presents the odds ratios of reporting an honour killing compared to a male suicide. Model 2 presents the odds ratios of reporting a kin-perpetrated honour killing compared to a non-kin-perpetrated honour killing. ***p < 0.001, **p < 0.01, *p < 0.05, ∙ *p* < 0.1. Variables are standardized meaning the odds ratios correspond to a 1 standard deviation change in the variable

	Model 1 Outcome variable: 1 = honour killing 0 = male suicide	Model 2 Outcome variable: 1 = kin-perpetrated honour killing 0 = non-kin-perpetrated honour killing
	OR (95% CI)	OR (95% CI)
Sex ratio 15−49	0.74∙ (0.55−1.00)	0.80** (0.68−0.93)
Population	0.71 (0.45−1.12)	0.95 (0.76−1.18)
Night light luminosity	1.56∙ (0.98−2.47)	1.12 (0.90−1.39)
Police stations per division	0.95 (0.70−1.28)	0.99 (0.88−1.12)
Proportion of the population living in an urban area	1.35 (0.89−2.07)	1.19∙ (0.97−1.47)
Proportion of property that is owned by women	0.83 (0.60−1.16)	0.88 (0.73−1.05)
Sex difference in literacy	0.93 (0.69−1.25)	1.14 (0.96−1.36)
Observations	18,212	5902
Districts	78	60
Divisions	25	24
Provinces	4	4

Given that so far we have deduced that an increasingly male-biased sex ratio may be associated with reports of (1) male suicide over and above all honour killings and (2) honour killings perpetrated by non-kin over and above those perpetrated by kin, we can further check this by making two additional predictions: that sex ratio should have a stronger association with male suicide when we use only kin-perpetrated honour killings as the comparison group and a weaker or absent association when we use non-kin-perpetrated honour killings as the comparison group. This is indeed what we find (Supplementary Information, Table S4).

We include a number of robustness checks. Results are indistinguishable if we use the proportion of the population aged 15–49 that is male instead of the ratio (number of men per 100 women) (Table S5). Increasing the minimum report threshold for a district to be included in the analysis to 40 results in loss of significance for Model 1 but not Model 2 (Table S6). Removal of outlier districts results in loss of significance for Model 1 but not Model 2 (Table S7). Model 1 is, however, robust to limiting the analysis to only reports from 2017, the same year as the census from which we calculated the sex ratio, and in fact the strength of the relationship increases (Table S8). We are unable to run this robustness check for Model 2 due to sample size. Removing all control variables results in a large decrease in effect size and loss of significance for Model 1 but not for Model 2 (Table S9). This is expected given that both the reporting bias and drivers of the two types of honour killing are going to be significantly more similar than those for honour killings compared to male suicides, and therefore the comparison of the two will be less effected by confounders.

## Discussion

Femicide by partners/ex-partners and male suicides are among the most common causes of violent death for young adults worldwide (Campbell et al., 2007; Pitman et al., 2012), whereas femicide at the hands of natal family members (such as siblings, parents or cousins) is less common, but is more concentrated in the Middle East, North Africa, and parts of South Asia, and is associated with honour-based violence (Gill, 2017). In general, our results suggest that non-kin femicide is associated with a male-biased sex ratio, but kin-perpetrated femicide is less so. Honour killings of young women by their natal families may be driven more by factors at the family rather than the population level.

We expected that a male-biased sex ratio should be associated with (1) reduced reports of all honour killings/femicide relative to male suicide and (2) reduced reports of kin-perpetrated honour killings relative to non-kin-perpetrated honour killings. Instead, we found the opposite. Reports of male suicides appear to be higher in male-biased regions (compared to all reports of honour killings), as are reports of non-kin-perpetrated honour killings (compared to kin-perpetrated). However, the former was not robust to more stringent model specifications. Rather than lending support to the notion that the relative scarcity of women within a population is associated with an increase in their bargaining power or perceived value, these results instead lend support to the notion that an excess of males may increase male–male competition, in turn leading to violence against women.

In our sample, the majority of non-kin femicides were perpetrated by husbands/exes and in-laws. From an evolutionary perspective, higher rates of non-kin femicide in male-biased areas may be evidence of increased mate guarding by intimate partners (and in-laws) where women are scarce and partners are difficult to replace. In Pakistan, women often live with their mothers-in-law and conflict with in-laws is a frequently cited reason for domestic violence (Ali & Gavino, 2008). From an inclusive fitness perspective, in-laws have a vested interest in the behaviour of their daughters-in-law, given that they are the mothers of their grandchildren. Intimate-partner violence has been proposed as a behaviour to deter female infidelity and prevent women from leaving a relationship (Buss & Duntley, 2011). Theoretically consistent with this, intimate-partner violence often arises from feelings of jealousy or suspicions of infidelity, diminishing with age as women reach menopause (Kaighobadi et al., 2008; Wilson & Daly, 1996; Wilson et al., 1995). Femicide may be a consequence of mate guarding related intimate-partner violence given that the two principal risk factors for femicide are previous domestic violence and estrangement (Campbell et al., 2007). Supporting these theories, associations between male-biased sex ratios and intimate-partner violence and femicide have been found in India, the USA, and six Asian and Pacific countries (Amaral & Bhalotra, 2017; Avakame, 1999; Bose et al., 2013; D’Alessio & Stolzenberg, 2010; Diamond-Smith et al., 2018).

There were also many non-kin honour killings perpetrated by ‘others’ that included acquaintances and neighbours, which could require a different kind of theoretical explanation than that outlined above. Some propose that merely having a larger proportion of unmarried men in an area increases all forms of violence, because it is usually single men who are responsible for the largest share of violent offending (Daly & Wilson, 1988). It’s also possible that some of the ‘other’ perpetrators were intimate partners, or those who had been rejected in attempts to form relationships. Although many of the headlines in the data set are missing, some reports of honour killings perpetrated by the other category followed the rejection of a marriage proposal.

That kin perpetrated honour killings did not appear to vary with the sex ratio is surprising, given that murdering one’s own female kin would appear highly maladaptive under any circumstances, let alone when women are scarce. Although we cannot entirely rule out that kin-perpetrated honour killings do vary with the sex ratio, given that our analytical results are all in comparison to another group, it is still surprising that this association would appear to be minimal compared to male suicide and non-kin-perpetrated honour killings. One possibility is that the likelihood that a woman is accused of dishonourable behaviour and the degree to which a family is under pressure from the community to punish dishonourable behaviour is independent of the sex ratio. For example, when a woman is accused of acting dishonourably families can face ostracism from the community until honour is restored (D’Lima et al., 2020; Gill, 2017). By virtue of being connected with a dishonourable woman, marriage and job opportunities as well as the overall status of her siblings and extended family are negatively affected. In these circumstances the cost to the family of not punishing, in terms of the loss of status and access to economic resources, may still outweigh the cost of punishing, even in male-biased areas. From an evolutionary perspective, there are many other contexts worldwide in which family members may discriminate against some offspring in order to promote the success of other offspring, for example in the case of urging offspring into religious celibacy (Micheletti et al., 2022; Micheletti & Mace, 2024) or of infanticide (Hrdy, 2009). So, it is possible that costs and benefits deriving from competition within the family can drive behaviours that appear maladaptive.

Why might a male-biased sex ratio also associate with increased reports of suicide? Depression often arises from adversity and the failure to achieve key social goals like marriage or career success (Nesse, 2000; Stieglitz et al., 2015).

As the sex ratio reflects the marriage market (Angrist, 2002), a male-biased sex ratio may increase competition for brides, leaving some unmarried, which could lead to depression and suicide. This has been documented in Japan (Kuroki, 2014) and the USA (Snopkowski & Turner, 2023), with the number of unmarried men mediating the association in the USA. Similarly in China, higher rates of depression are found among unmarried men living in male-biased areas (Zhou & Hesketh, 2017). These are of course not the only explanations for suicide. However, we caution against overinterpreting this result, because it was not robust to other model specifications and may be influenced by omitted variables. In Pakistan, male-biased sex ratios will be in large part driven by sex-biased economic migration (Hamid, 2010). These economic migrants are often (although not always) from poorer sections of society, and maintain strong links to their place of origin, with many leaving behind wives and children (Mansuri, 2006). This is counter to the idea that the association between sex ratio and suicide is being driven by unmarried men. Rather, depression may be endogenous to migration in a context where principally poor men are having to migrate in order to send remittances back home. In this case poverty, a well-known risk factor for depression and suicide, could be driving the association we see between sex ratio and suicide. More demographic information on the individuals would be needed to tease apart these different pathways.

### Limitations

This study examines associations between aggregate-level sex ratio at the district-level and individual-level outcomes. Aggregate-level sex ratio data have been criticized due to theoretical issues with the appropriateness of the level of aggregation, most notably whether it constitutes a meaningful measure of an individual’s local marriage market (Pollet et al., 2017). Additionally, associations found at the aggregate level can often be reversed to those found at a lower level (Filser et al., 2021). Here we use the lowest level of aggregation possible. Whether district represents a meaningful measure of the ‘local’ mating market is debateable. Additionally, the mechanism via which the sex ratio effects behaviour will itself be affected by how people perceive the sex ratio. Indeed, perception of the sex ratio can bear little relation to the actual sex ratio (Filser & Preetz, 2021; Gilbert et al., 2016). Second, spatial clustering between aggregated units can lead to spurious associations driven by broader regional dynamics (Pollet et al., 2017). We partially address this issue by performing multi-level models, clustering our data at the district, division and region levels to address that statistical relationships could be driven by non-independence of data points.

The fundamental limitation of this research is that we rely on newspaper reports of violence, which suffers from a high degree of selection bias. However, no other data set of honour-based violence exists, and newspaper reports are currently the only means by which any kind of reliable estimate of honour-based violence is generated.

## Conclusion

In sum, we use a novel data set of newspaper reports of violence against women and suicide in Pakistan to show that male-biased sex ratios may be associated with honour killings perpetrated by non-kin such as husbands and in-laws. Further research is needed to consolidate these results, particularly the relationship between the sex ratio and male suicide.

## Supporting information

Campbell et al. supplementary material Campbell et al. supplementary material

## Supplementary material

The supplementary material for this article can be found at https://doi.org/10.1017/ehs.2025.10003.

## Research transparency and reproducibility interest

Data availability is at the discretion of the Human Rights Commission of Pakistan.
