# Supplementary material for: Skewed sex ratios and violence against women in Pakistan
Source: Evol Hum Sci. 2025 Jul 1;7:e18. doi: 10.1017/ehs.2025.10003 (PMC12344599; doi:10.1017/ehs.2025.10003)

**Supplementary Information for “Skewed sex ratios and violence against women in Pakistan”**

Olympia L K Campbell, Maheen Pracha, Ruth Mace

Table of Contents

[Table S1: List of HRCP offices and the newspapers scanned for human rights abuses 3](#_Toc175671914)

[Table S2: Keywords used by the HRCP for particular categories of human-rights abuse 4](#_Toc175671915)

[Table S3: Total number of all media reports of honour killings and suicide across the 7 provinces 5](#_Toc175671916)

[Table S4: Comparing male suicide to kin and non-kin honour killings separately. Note that we have reversed the cases and controls to make male suicide the case. 6](#_Toc175671917)

[Table S5: Using proportion of the population aged 15-49 that is male instead of the sex ratio 7](#_Toc175671918)

[Table S6: Including only districts that had a combined number of at least 40 reports 8](#_Toc175671919)

[Table S7: Removing outlier districts 9](#_Toc175671920)

[Table S8: Including only media reports from the year 2017 10](#_Toc175671921)

[Table S9: Removing all controls 11](#_Toc175671922)

[Figure S1: Correlation between the log of all reports of male suicide per district and the log of all reports of honour killings per district 12](#_Toc175671923)

[Figure S2 – Correlation between the sex ratio of those aged 15-49 (number of men per 100 women) and the percentage of a district’s population living in an urban area 13](#_Toc175671924)

## Table S1: List of HRCP offices and the newspapers scanned for human rights abuses

| Province | Office | Newspaper |
| --- | --- | --- |
| **Punjab** | Lahore | Dawn  Dawn (Metro Islamabad)  Nation  Express tribune  The News  Jang  Nawaiwaqt  Daily express  Duniya  Voicepk.net  Friday Times - Naya Daur  Dawn Prism |
|  | Multan | Jang (Metro)  Express (Metro)  Dunya (Metro) |
| **Sindh** | Karachi | Dawn (Metro)  Express (Metro) |
|  | Hyderabad | Pahenji  Kavish |
| **Balochistan** | Quetta | Jang (Metro)  Daily Intekhab |
|  | Turbat | Daily Intekhab (Metro) |
| **Khyber Pakhtunkhwa** | Peshawar | Mashriq  Aaj  Express (Metro) |
| **Gilgit-Baltistan** | Gilgit | Ausaf  Baad-e-Shimal |

## Table S2: Keywords used by the HRCP for particular categories of human-rights abuse

| Main Category | Subcategory | Keywords |
| --- | --- | --- |
| **Gender-based violence** | Harmful custom practices | Honor killing, murder, Karo-kari, Wani, swara, Kala-Kali, forced marriages, underage marriages, acid crime, dowry-related violence, son preference |
| **Crimes** | Suicide | Suicide attempt, self-immolation, reasons (poverty, unemployment, psychological violence, mental health, etc.) |

## Table S3: Total number of all media reports of honour killings and suicide across the 7 provinces

|  | Honour killing | Suicide |
| --- | --- | --- |
| Punjab* | 5166 | 12854 |
| Sindh | 1926 | 4480 |
| KPK | 1255 | 1216 |
| Balochistan | 308 | 141 |
| Gilgit Baltistan | 7 | 9 |
| Azad Jammu Kashmir | 6 | 4 |
| FATA | 7 | 0 |

*includes Islamabad.

KPK = Khyber Pakhtunkhwa, FATA = Federally Administered Tribal Areas

N.B. these numbers do not correspond to the numbers in Table 1 in the main manuscript as they include female suicides as well as reports where the relationship between victim and perpetrator is not known.

## Table S4: Comparing male suicide to kin and non-kin honour killings separately. Note that we have reversed the cases and controls to make male suicide the case.

|  | **Model 1**  Outcome variable:  1 = male suicide  0 = kin perpetrated honour killing | **Model 2**  Outcome variable:  1 = male suicide  0 = non-kin perpetrated honour killing |
| --- | --- | --- |
|  | OR (CI) | OR (CI) |
| Sex ratio 15-49 | 1.57*  (1.00-2.47) | 0.80  (0.58-1.11) |
| Population | 1.41  (0.86-2.33) | 0.74  (0.45- 1.20) |
| Night light luminosity | 0.67  (0.40-1.11) | 1.50  (0.91-2.48) |
| Police stations per division | 1.14  (0.67-1.96) | 0.88  (0.62-1.24) |
| Proportion of the population living in an urban area | 0.69  (0.43-1.09) | 1.23  (0.78-1.93) |
| Proportion of property that is owned by women | 1.39  (0.94-2.04) | 0.83  (0.58-1.18) |
| Sex difference in literacy | 1.11  (0.74-1.65) | 0.82  (0.60-1.14) |
| Observations | 12870 | 14491 |
| Districts | 71 | 74 |
| Divisions | 24 | 24 |
| Province | 4 | 4 |

## Table S5: Using proportion of the population aged 15-49 that is male instead of the sex ratio

|  | **Model 1**  Outcome variable:  1 = honour killing  0 = male suicide | **Model 2**  Outcome variable:  1 = kin perpetrated honour killing  0 = non-kin perpetrated honour killing |
| --- | --- | --- |
|  | OR (CI) | OR (CI) |
| Proportion of men 15-49 | 0.74*  (0.54-0.99) | 0.80**  (0.69-0.94) |
| Population | 0.71  (0.45-1.13) | 0.95  (0.76-1.19) |
| Night light luminosity | 1.56•  (0.98-2.45) | 1.11  (0.84-1.38) |
| Police stations per division | 0.94  (0.69-1.28) | 0.99  (0.88-1.12) |
| Proportion of the population living in an urban area | 1.36  (0.89-2.08) | 1.19  (0.96-1.47) |
| Proportion of property that is owned by women | 0.82  (0.58-1.15) | 0.87  (0.73-1.05) |
| Sex difference in literacy | 0.92  (0.69-1.25) | 1.15  (0.96-1.36) |
| Observations | 17900 | 5513 |
| Districts | 67 | 46 |
| Divisions | 24 | 21 |
| Province | 4 | 4 |

The district level proportion of men is calculated as:

number of men aged 15-49 / (number of men aged 15-49 + number of women aged 15-49)

## Table S6: Including only districts that had a combined number of at least 40 reports

|  | **Model 1**  Outcome variable:  1 = honour killing  0 = male suicide | **Model 2**  Outcome variable:  1 = kin perpetrated honour killing  0 = non-kin perpetrated honour killing |
| --- | --- | --- |
|  | OR (CI) | OR (CI) |
| Sex ratio 15-49 | 0.83  (0.51-1.16) | 0.84*  (0.72-0.97) |
| Population | 0.6465194•  (0.41-1.02) | 0.98  (0.79-1.22) |
| Night light luminosity | 1.55•  (0.95-2.51) | 1.08  (0.86-1.36) |
| Police stations per division | 0.99  (0.73-1.35) | 0.98  (0.88-1.10) |
| Proportion of the population living in an urban area | 1.19  (0.77-1.84) | 1.08  (0.87-1.34) |
| Proportion of property that is owned by women | 0.94  (0.63-1.40) | 0.90  (0.75-1.09) |
| Sex difference in literacy | 0.88  (0.64-1.20) | 1.06  (0.87-1.31) |
| Observations | 17900 | 5513 |
| Districts | 67 | 46 |
| Divisions | 24 | 21 |
| Province | 4 | 4 |

We include only districts that recorded at least 40 combined reports of male suicides and honour killings for Model 1, and 40 reports of honour killings in which the relationship between the victim and perpetrator was known for Model 2.

## Table S7: Removing outlier districts

Implausible outlier districts are defined as those where the district-level proportion of male suicides (male suicides/male suicides + all honour killings for Model 1) and non-kin perpetrated honour killings (non-kin/non-kin + kin perpetrated honour killings for Model 2)) is either greater than 0.85 or less than 0.15.

|  | **Model 1**  Outcome variable:  1 = honour killing  0 = male suicide | **Model 2**  Outcome variable:  1 = kin perpetrated honour killing  0 = non-kin perpetrated honour killing |
| --- | --- | --- |
|  | OR (CI) | OR (CI) |
| Sex ratio 15-49 | 0.90  (0.70-1.15) | 0.81**  (0.70-0.93) |
| Population | 0.75  (0.52-1.07) | 0.95  (0.77-1.17) |
| Night light luminosity | 1.36•  (0.96-1.93) | 1.10  (0.89-1.36) |
| Police stations per division | 0.77*  (0.63-0.94) | 0.98  (0.88-1.08) |
| Proportion of the population living in an urban area | 1.14  (0.80-1.61) | 1.19•  (0.97-1.45) |
| Proportion of property that is owned by women | 0.95  (0.72-1.26) | 0.89  (0.75-1.06) |
| Sex difference in literacy | 0.93  (0.75-1.15) | 1.17•  (0.99-1.37) |
| Observations | 16536 | 5856 |
| Districts | 69 | 58 |
| Divisions | 23 | 23 |
| Province | 4 | 4 |

## Table S8: Including only media reports from the year 2017

|  | **Model 1**  Outcome variable:  1 = male suicide  0 = kin perpetrated honour killing |
| --- | --- |
|  | OR (CI) |
| Sex ratio 15-49 | 0.545**  (0.36-0.84) |
| Population | 0.89  (0.44-1.81) |
| Night light luminosity | 2.14*  (1.15-4.00) |
| Police stations per division | 0.96  (0.69-1.34) |
| Proportion of the population living in an urban area | 1.12  (0.58-2.17) |
| Proportion of property that is owned by women | 0.69  (0.36-1.34) |
| Sex difference in literacy | 0.69  (0.43-1.10) |
| Observations | 2392 |
| Districts | 41 |
| Divisions | 18 |
| Province | 3 |

Limits the media reports included in the analysis to only those from the year 2017 since the the sex ratio data is taken from the 2017 Pakistani census

## Table S9: Removing all controls

|  | **Model 1**  Outcome variable:  1 = honour killing  0 = male suicide | **Model 2**  Outcome variable:  1 = kin perpetrated honour killing  0 = non-kin perpetrated honour killing |
| --- | --- | --- |
|  | OR (CI) | OR (CI) |
| Sex ratio 15-49 | 0.95  (0.79-1.14) | 0.84**  (0.76-0.93) |
| Observations | 18312 | 5940 |
| Districts | 79 | 61 |
| Divisions | 25 | 24 |
| Province | 4 | 4 |

## Figure S1: Correlation between the log of all reports of male suicide per district and the log of all reports of honour killings per district


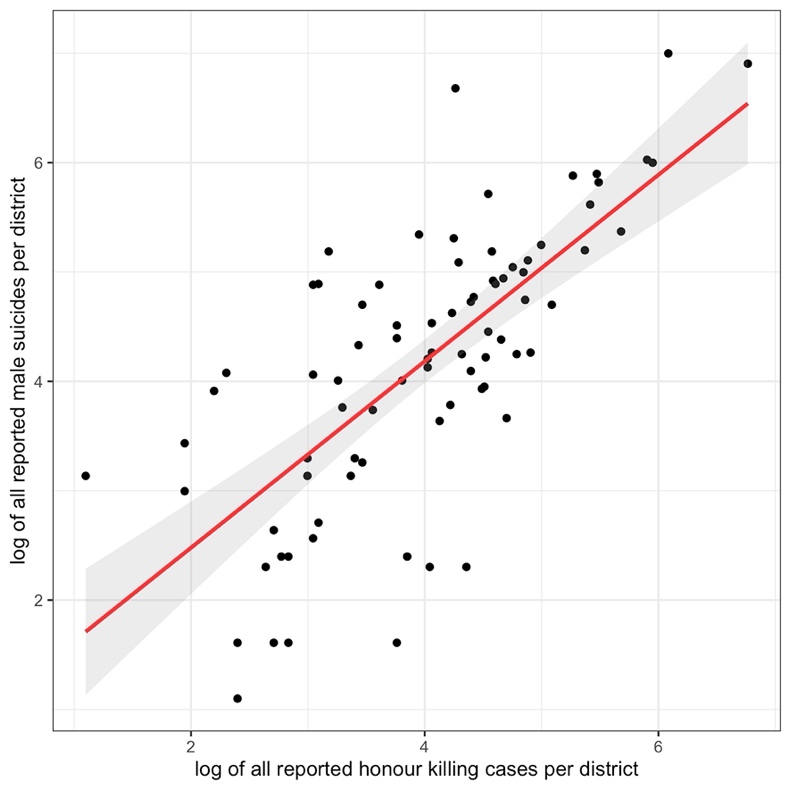


## Figure S2 – Correlation between the sex ratio of those aged 15-49 (number of men per 100 women) and the percentage of a district’s population living in an urban area


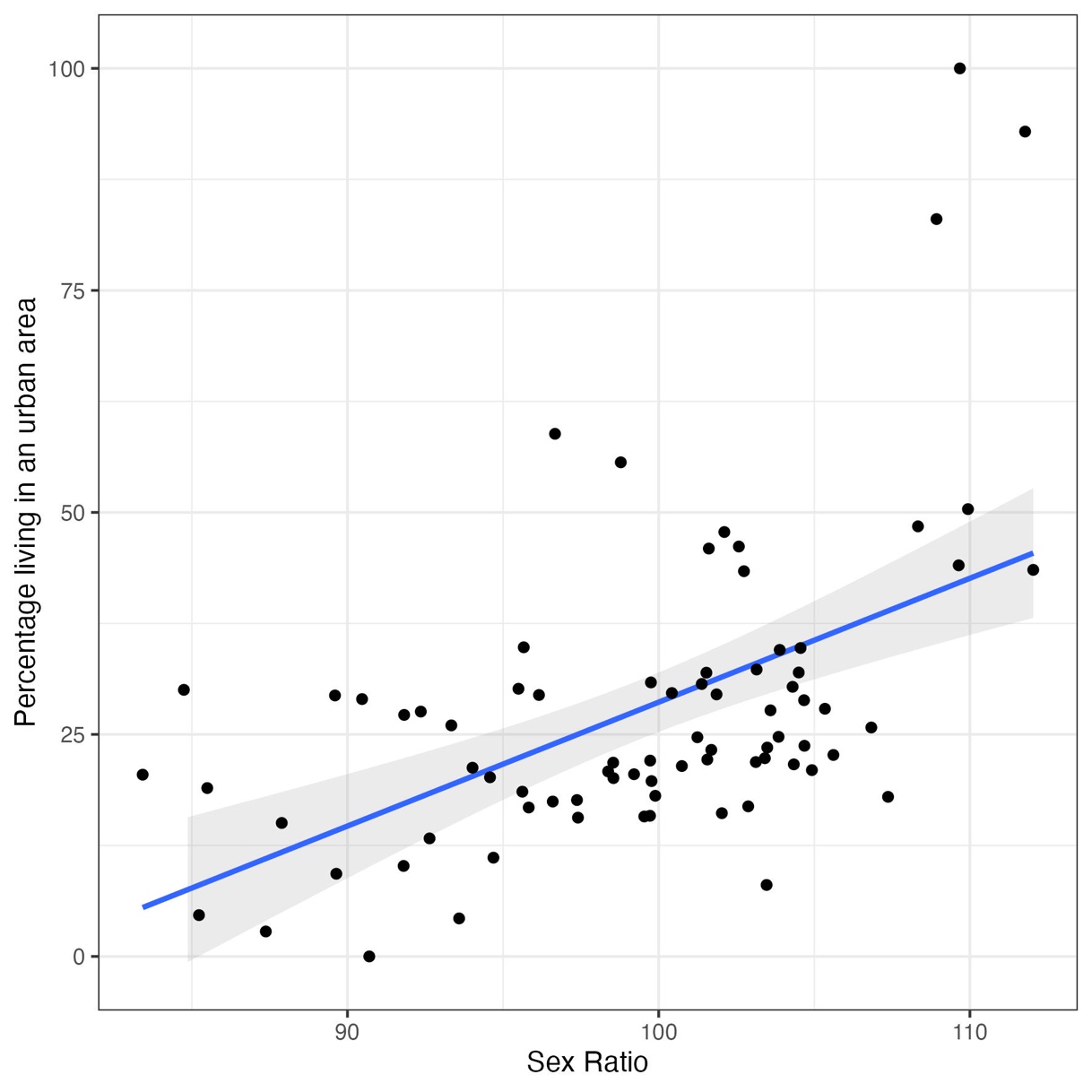

Supplement: Campbell et al. supplementary material [file S2513843X25100030sup001.docx]
